# Popliteal endarterectomy at the leg level with venous enlargement patch: about 14 cases

**DOI:** 10.1093/jscr/rjad398

**Published:** 2023-07-07

**Authors:** Hamid Channane, A Shaporov, A Sandica, S Atay, I Snopok, T Frunza, R Viebahn

**Affiliations:** Department of General and Vascular Surgery, Ruhr University, Bochum, Germany; Department of General and Vascular Surgery, Ruhr University, Bochum, Germany; Department of General and Vascular Surgery, Ruhr University, Bochum, Germany; Department of General and Vascular Surgery, Ruhr University, Bochum, Germany; Department of General and Vascular Surgery, Ruhr University, Bochum, Germany; Department of General and Vascular Surgery, Ruhr University, Bochum, Germany; Department of General and Vascular Surgery, Ruhr University, Bochum, Germany

**Keywords:** popliteal endarterectomy, venous patch plasty, popliteal PTA

## Abstract

The incidence of lesions of the popliteal artery below the knee constitutes one of the greatest problems in revascularization of the lower limb. Firstly, this segment constitutes the departure of the leg tripod, decisive crossroads for a subsequent endovascular intervention. On the other hand, it constitutes a fairly used relay point in the event of an indication for a pedal bypass. It is assumed that the performance of a popliteal endarterectomy with an enlargement by medial approach in patients with a localized lesion at this level constitutes an effective therapeutic approach and can facilitate any gesture of crural bypass or endovascular dilation later. We present a retrospective review of all patients who underwent popliteal endarterectomy with venous patch plasty for localized popliteal disease in our institution over the past 3 years.

## INTRODUCTION

In recent years, along with changes in lifestyle and aging, the incidence of complex multilevel or diffuse peripheral arterial disease has increased significantly. This also contributed to the increase in complexity of the therapeutic management, given the comorbidities of the patients. The choice of first-line intervention is often difficult in the presence of tibiopopliteal disease as well as multiple comorbid conditions like coronary arterial disease and renal insufficiency. Several procedures like endarterectomies, bypass grafting and endoluminal procedures are often used in combination with address these complex lesions. Thromboendarterectomy (TEA), mostly of the femoral region, was the procedure of choice for patients with peripheral arterial occlusive disease (PAOD) [1, 2]. With the advances in bypass techniques and availability of prosthetic grafts, there is a marked decline in the number of surgeons performing this technically demanding procedure [1].

In 1946, the Portuguese surgeon Dos Santos did the first TEA through two arterectomies, referring to it as disobliteration [1]. Leriche termed the removal of an obstructing thrombus as well as diseased arterial intima as thromboendarterectomy (TEA). It has been shown that excellent results can be achieved when the technique was precisely performed, especially in the popliteal artery segments [2].

Over the last few years, there has been a significant shift towards lower limb revascularization using endoluminal techniques [3]. However, endoluminal techniques alone are often unable to salvage limbs that exhibit tissue loss. In multilevel arterial occlusion especially with involvement of tibio-popliteal segment, endarterectomy is a viable option as we were able to show by the results in our series of patients.

## MATERIALS AND METHODS

A retrospective study of twelve consecutive patients with complex arterial occlusive disease with involvement of the popliteal artery in the infraguneal segment was made in our surgical department. The patients have been operated during a period of three years, from 2020 to 2023. A large popliteal endarterectomy was done in all these cases in addition to venous patching. Cases of femoropopliteal disease involving also the femoral artery were excluded from the study. Demographic data, comorbidities, laboratory data, angiographic anatomic data and clinical outcome were recorded. All patients underwent a preoperative assessment including CT-angiography. Angioplasty was attempted in all patients before surgical intervention. Patency was assessed radiologically 6 weeks after operation. Patients had follow-up appointments at intervals of 6 weeks, 3 months, 6 months, and a year after surgery.

**Figure 1:**
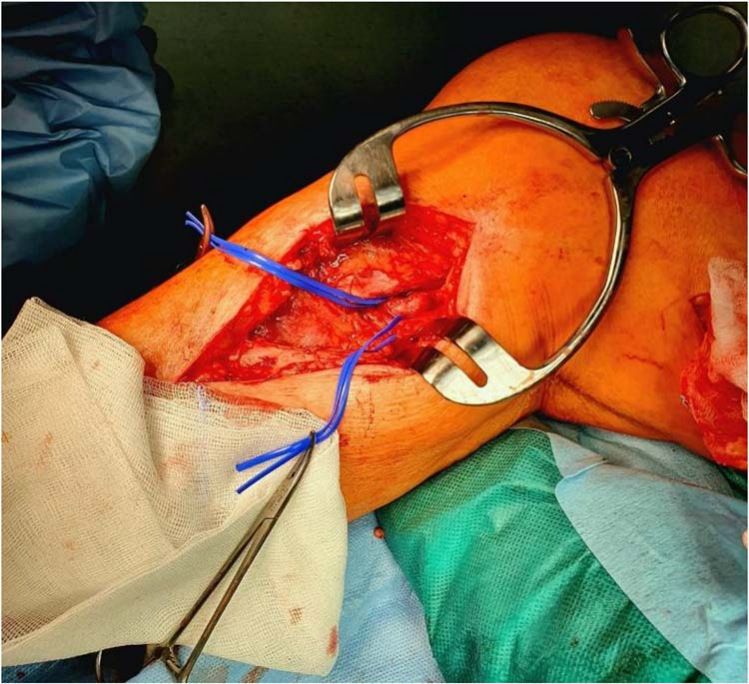
Venous patching after a popliteal endarterectomy.

Surgery of the popliteal artery was performed under general anesthesia by median approach of the infraguneal segment of the popliteal artery as well as the leg trifurcation. After preparation and putting in lake of all the arterial structures, a heparin bolus of 5000 IU was administered intravenously at the start of the procedure and the ACT was maintained above 250 s. Subsequently, a segment of at least 10 cm of the internal saphenous vein was prepared and removed to ensure the realization of an enlargement patch (Fig. 1). After arterial clamping, an arteriotomy is performed along the popliteal artery taking a caudal direction along the tibio-peroneal trunk. Subsequently, an endarterectomy is performed including the tibio-peroneal trunk and the proximal part of the anterior tibial artery before it crosses the interosseous membrane.

## RESULTS

**Figure 2:**
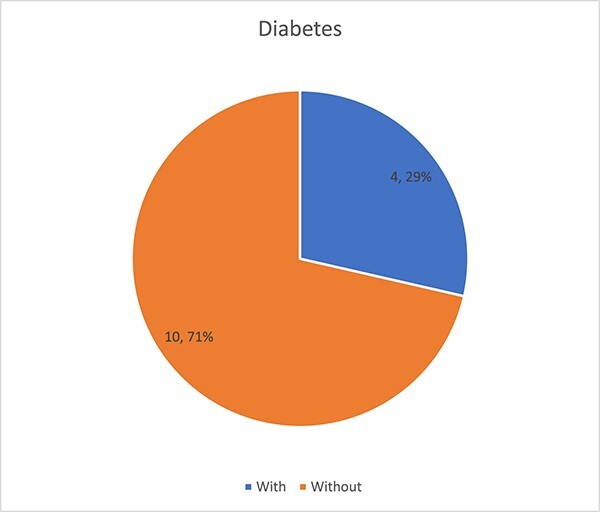
Diabetes as a risk factor.

**Figure 3:**
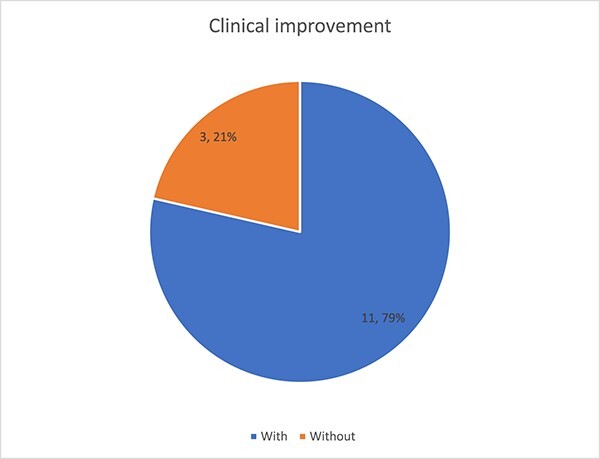
Clinical improvement after treatment.

A total of 14 patients (9 men and 5 women) underwent popliteal endarterectomy. The mean age was 66.1 years, with a mean follow-up period of 13 months (range: 2–26 months), where 64% of patients were male. Four patients were treated for activity-limiting claudication (<100 yards), whereas three patients were treated for ischemic rest pain and seven patients were treated for ischemic ulcerations. The procedural success rate was 100% without mortalities or in-hospital morbidities. Hypercholesterolemia was found in almost 71% of patients in our series, while diabetes was a risk factor in four patients or 29% of all patients (Fig. 2). Regarding smoking, it was found in 64% of our patients. Symptomatic resolution was achieved in 11 patients, while two patients had stagnation of symptoms and one patient had worsening symptoms and required arteriography with dilation of the leg arteries (Fig. 3).

## DISCUSSION

Peripheral arterial disease correlates strongly with risk for major cardiovascular events and often coexists with coronary and cerebrovascular disease [1–4]. Because the prevalence of PAD increases progressively with age, it is a major health problem in elderly.

Most patients with lesions in the AFS also have stenoses further distally, in the motor segment of the popliteal artery, which also have to be treated if symptomatic [5]. Anatomical conditions, such as a narrow vascular lumen, increased mechanical stress on the arteries due to movement of the knee joint, slower blood flow and more stenosis in the distal outflow area could pose an additional challenge for the treatment and outcome of popliteal artery lesions. [10].

Most current investigations are limited to vascular sections of the AFS above the knee joint and thus above the motion segment [6, 7]. Thromboendarterectomy (TEA) was more frequently performed in the femoral area in patients with peripheral arterial occlusive disease (PAD) until the introduction of bypass techniques and availability of prosthetic grafts. TEA had been discouraged due to perioperative thrombosis, increased morbidity and rate of amputation in advantage to endovascular practices [1, 4].

More recently, many authors had established the role of popliteal endarterectomy in chronic critical limb ischemia in combination with other procedures in multilevel arterial obstructive disease [3, 4, 8]. Hybrid endovascular and open surgical revascularization procedures might be of benefit because of its less invasive character compared to other surgical procedures, lack of extensive venous graft material, and the ability to overcome long-segment arterial obstructions [4]. However, diabetes, renal insufficiency and chronic limb ischemia could compromise its long-term patency.

The effectiveness of popliteal TEA is confirmed in the symptomatic improvement and salvage of the limb at lower costs, when the indications and the accuracy of surgical techniques are respected [6]. It offers advantages for the surgical treatment of steno-obstructive lesions of the crural trifurcation; this anatomical region being a key area for subsequent endovascular recanalization of possible arterial lesions or pedal bypasses in the leg.

In patients with limb ischemia, the quality of life may also be improved by physical training, vasoactive medicaments and optimal management of concomitant diseases [9]. Aspirin and secondly clopidogrel are suited for secondary prevention of arteriosclerosis not only in the extracranial, but also in the peripheral vascular region and improve long-term outcome by reducing the incidence of restenosis especially after local thrombolysis. Various pharmacologic agents may be used to facilitate healing and therefore improve the results of endarterectomy like heparin, ACE inhibitors and growth factor antagonists.

In our series, the majority of our patients were elderly diabetic with stable ischemic heart disease and tissue loss at presentation with other comorbidities and could be operated under spinal anesthesia with minimal postoperative morbidities. These patients remained pain free and showed good long-term results.

Because of low cost, use of autogenous vascular reconstruction, no prosthetics use, low risk of infection, less morbidities, popliteal TEA will assume an increasingly important role in complex peripheral arterial occlusive disease. To conclude, popliteal TEA constitutes a therapeutic option, which can play a crucial role in the revascularization of complex PAD, and would make endovascular gestures and/or bypasses possible or at least more simple.
